# Intracorporeal circular-stapled gastroduodenostomy for billroth I reconstruction in total laparoscopic distal gastrectomy

**DOI:** 10.3389/fsurg.2025.1635611

**Published:** 2025-09-19

**Authors:** Ying Yang, Linsen Zhou, Jing Zhuang, Yizhou Sun, Yuemei Ding, Haohai Jiang

**Affiliations:** Department of General Surgery, The Yancheng Clinical College of Xuzhou Medical University & The First People’s Hospital of Yancheng, Yancheng, Jiangsu, China

**Keywords:** total laparoscopic distal gastrectomy (TLDG), circular-stapled gastroduodenostomy, purse-String suture clamp (PSC), multi-functional seal cap(MSC), gastric cancer

## Abstract

**Objectives:**

While laparoscopic distal gastrectomy (LDG) has gained acceptance for early gastric cancer, challenges persist in intracorporeal circular-stapled gastroduodenostomy during totally laparoscopic distal gastrectomy (TLDG). This study introduces a novel technique using a laparoscopic purse-string suture clamp (Lap-PSC) and multifunctional sealing cap (MSC) to simplify Billroth I circular-stapled anastomosis.

**Methods:**

A single-center retrospective analysis was conducted on 47 gastric cancer patients undergoing TLDG with Lap-PSC and MSC between September 2022 and June 2024. Surgical procedures involved D2 lymph node dissection, a 5 cm upper abdominal incision for specimen extraction, and intracorporeal circular-stapled anastomosis using Lap-PSC for duodenal purse-string suturing and MSC for pneumoperitoneum maintenance. Postoperative outcomes, complications, and anastomotic integrity were evaluated.

**Results:**

All 47 patients underwent successful TLDG with negative resection margins. Mean operative time was 148.3 ± 41.4 min. One patient (2.1%) experienced postoperative gastroparesis, and one patient (2.1%) experienced postoperative pneumonia. No anastomotic leaks, strictures, or perioperative deaths occurred. Median follow-up was 21.4 months (range: 13–34), with no recurrence or anastomosis-related complications.

**Conclusion:**

TLDG with Circular-Stapled Gastroduodenostomy for Billroth I anastomosis demonstrates technical feasibility and safety, combining the precision of open surgery with laparoscopic minimally invasive benefits. The technique simplifies intracorporeal circular-stapled anastomosis, avoids excessive tension, and may reduce ischemic risks associated with linear stapler methods.

## Introduction

According to GLOBOCAN 2022, gastric cancer is the fifth most common cancer and fifth leading cause of cancer deaths globally (968,350 new cases, 659,853 deaths), while in China, despite declining rates, it remains the fifth most common and third deadliest cancer, with 358,700 new cases and 260,400 deaths in 2022 (1, 2). The effective treatment for gastric cancer is surgical resection, including open surgery, laparoscopic surgery, and robotic surgery. Kitano et al. first reported a case of laparoscopic-assisted gastrectomy for early distal gastric cancer in 1994 (3). Since the publication of that report, this surgical approach has rapidly gained popularity in Eastern countries, particularly for the treatment of early gastric cancer (T1, any N, and M0). The JCOG0912 and KLASS-01 studies confirmed laparoscopic distal gastrectomy (LDG) as non-inferior to open distal gastrectomy (ODG) for early gastric cancer, while China's CLASS-01 trial demonstrated comparable 3-year disease-free survival, 5-year overall survival, and postoperative safety for LDG in advanced gastric cancer, supporting its recommendation in CSCO guidelines for experienced centers (4–8). Billroth Ⅰ anastomosis is a traditional operation for distal gastric reconstruction of the digestive tract, which is valid but less commonly used technique compared to Roux-en-Y, especially under laparoscopy. Circular-stapled anastomosis is routinely used in open surgery. However, transitioning from the use of a circular stapler in open surgery to a fully intracorporeal circular stapler represents a significant change in surgical practice for most gastrointestinal surgeons.

Based on our previous research on laparoscopic Purse-String Suture Clamp (Lap-PSC; WODELIPAI, Wuhan, HB, China) and a multifunctional sealing cap (MSC; WODELIPAI, Wuhan, HB, China) technology (9), our team has designed a novel laparoscopic circular-stapled for Billroth I anastomosis of the distal stomach. During a median follow-up period of 21.4 months, this surgical procedure has demonstrated satisfactory early postoperative results without any anastomotic-related complications. With these instruments and techniques, the standard circular-stapled gastroduodenostomy used in ODG can be applied in TLDG. Below are the results of our research to date.

## Methods

### Patients and techniques

From September 2022 to June 2024, 47 gastric cancer patients at our hospital underwent TLDG using the Lap-PSC device and the MSC technique, with all patients receiving intraluminal single-stapled gastroduodenostomy using a circular-stapler. The inclusion criteria were: preoperative imaging, endoscopy, and histological confirmation of gastric adenocarcinoma located in the lower part of the stomach, with no adjacent organ involvement or distant metastasis. None of the cases had received neoadjuvant therapy. The choice of surgical approach was based on the patient's personal decision after understanding the advantages and disadvantages of each method. All 47 patients signed written informed consent forms prior to surgery.

### Operative procedures

After general anesthesia induction, the patient was placed in a French position with legs separated. The camera port was positioned just below the umbilicus. Establish pneumoperitoneum and insert trocars using the five-port technique, with the left upper puncture site serving as the primary operating port.

**Figure 1:**
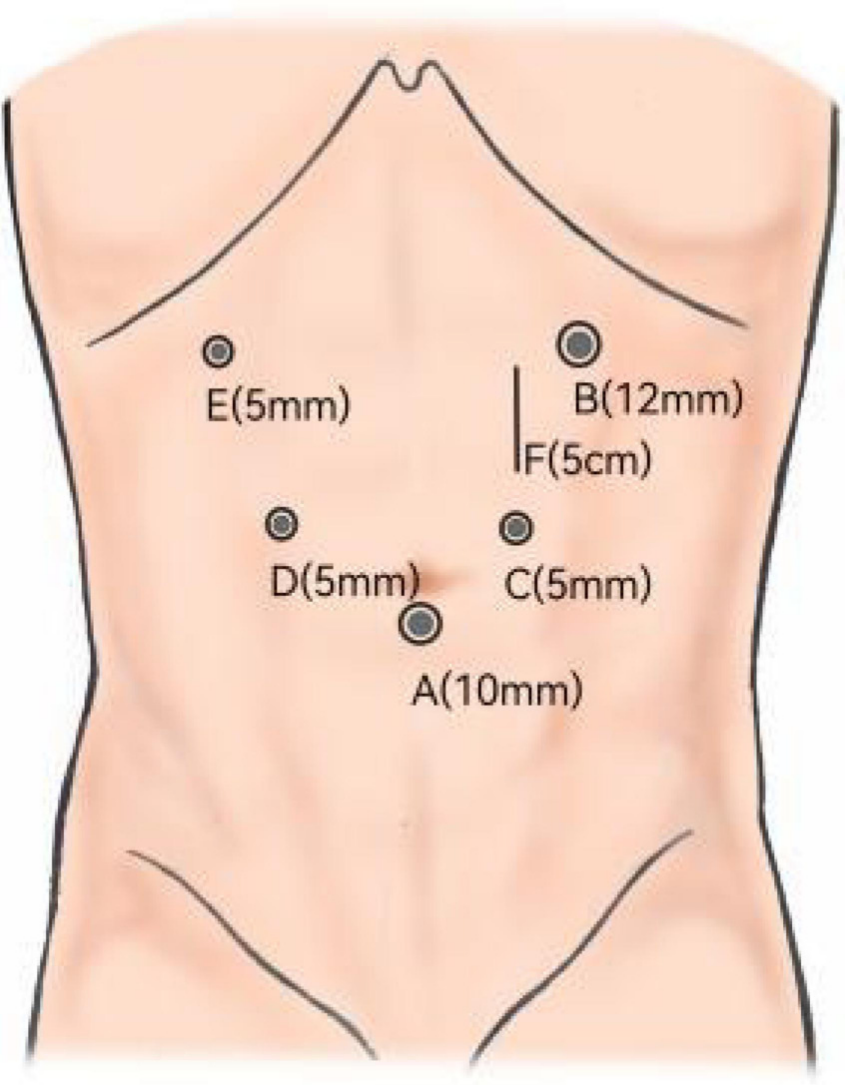
Trocar placement. **(A)** Camera port for laparoscopy. **(B)** Main operating port. **(C,D)** Assisted ports. **(F)** Abdominal incision approximately 5 cm for MSC.

**Figure 2:**
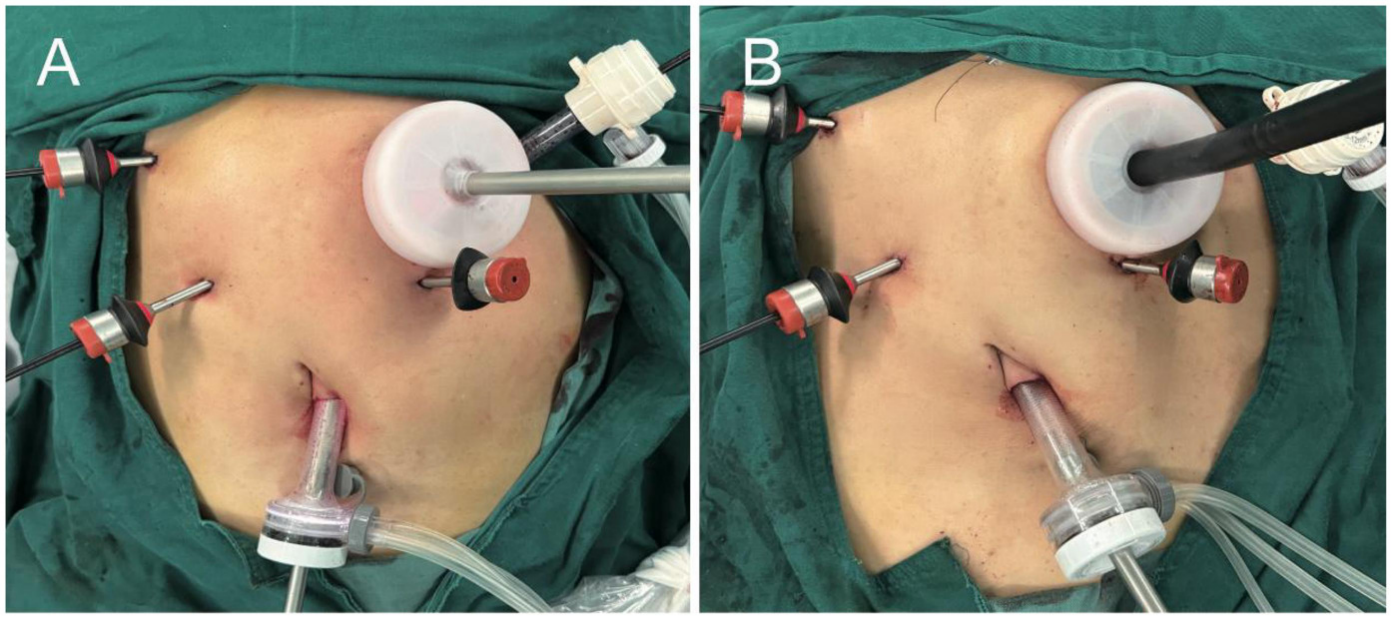
Photographs during the purse-string suture and gastroduodenostomy procedures. **(A)** Direct introduction of Lap-PSC into the abdominal cavity via the central opening on MSC. **(B)** Circular stapled gastroduodenostomy via the central opening on MSC.

**Figure 3:**
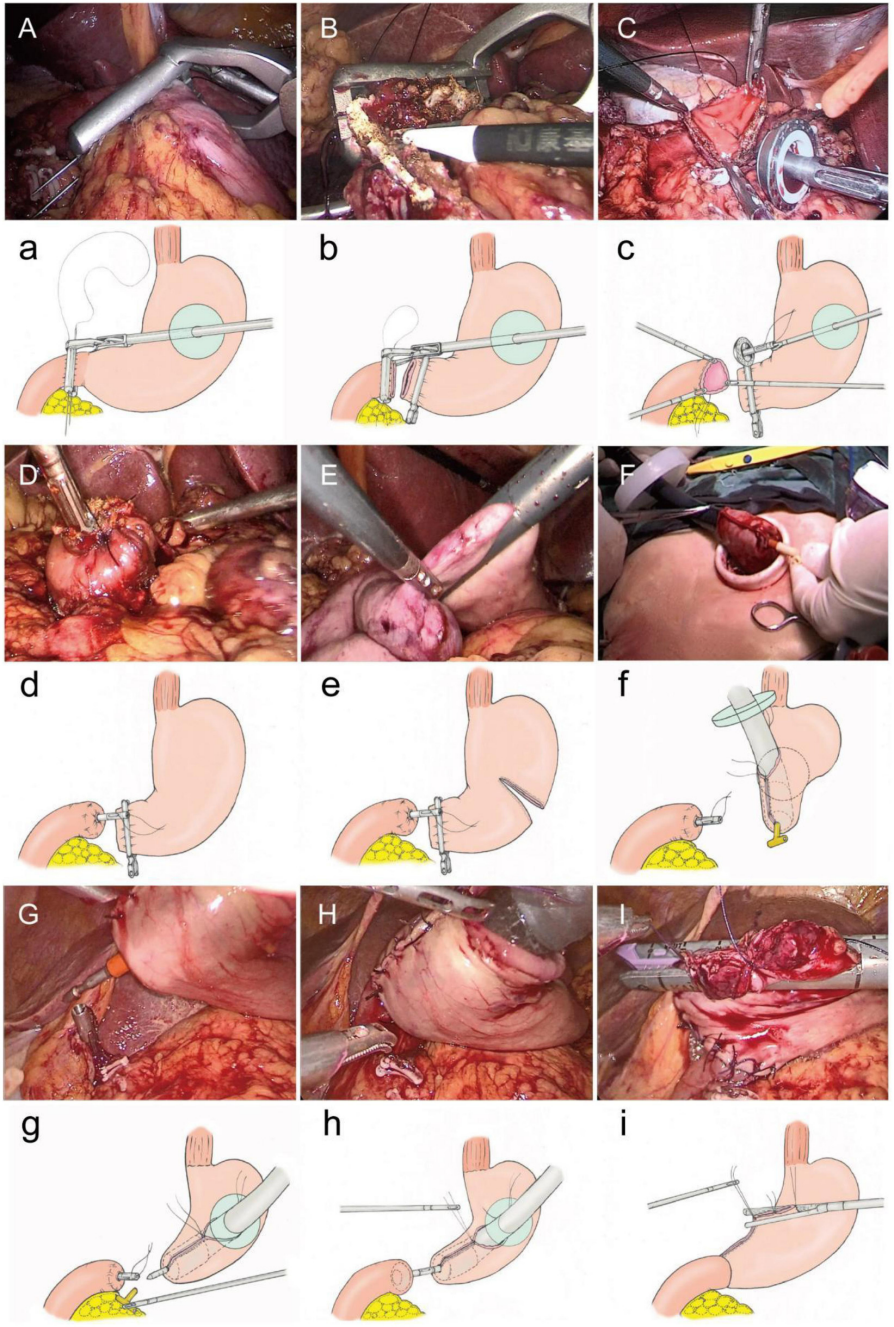
Operative procedure. **(A, a)** Lap-PSC insertion via MSC, purse-string needle placement. **(B, b**) Transect the duodenum. (**C, c)** Tract the duodenal stump in a triangular configuration and insert the anvil. **(D, d)** Tighten and tie the purse-string suture. **(E, e)** Transect stomach with linear cutting stapler along marked resection line. **(F, f)** Insert circular stapler into residual stomach (lesser curvature), rotate shaft to protrude tip at greater curvature, then insert an T- tube into the head of the circular stapler. **(G, g)** Rotate shaft, insert circular stapler via MSC, re-establish pneumoperitoneum, and remove T-tube. **(H, h)** Connect shaft to duodenal anvil, adjust anastomosis angle with traction sutures. **(I, i)** Close the opening on the lesser curvature side of the residual stomach using a linear cutting stapler via MSC.

After routine completion of D2 lymph node dissection of the distal stomach, make an incision of approximately 5 cm in the left upper abdomen (Figure 1). Protect the incision with an incision protector, and use this incision to insert the pretreated anvil (with the tail end of the anvil threaded through a small hole using silk thread, tied in a knot, and trimmed to about 5 cm), the Lap-PSC (Figure 2A), and the circular stapler (Figure 2B), as well as to remove the specimen. Sequentially pass the anvil and the Lap-PSC through the MSC (which is used in conjunction with the incision protector and features a small central hole to maintain pneumoperitoneum stability during intra-abdominal manipulation of the purse-string clamp and circular-stapler). Insert the Lap-PSC into the abdominal cavity through the central hole of the MSC, open the clamp jaws under laparoscopic guidance, clamp the duodenum 2–3 cm below the pylorus, insert the purse-string needle (Figures 3A,a), and occlude the proximal end with a blocking clamp. Use an electrocautery hook to transect the duodenum close to the Lap-PSC (Figures 3B,b). The surgeon and assistant subsequently tract the duodenal stump in a triangular configuration (Figures 3C,c), insert the anvil, and tighten and tie the purse-string suture (Figures 3D,d). Use linear cutting stapler to transect the stomach along the pre-marked resection line from the greater curvature to the lesser curvature (Figures 3E,e). Deflate the pneumoperitoneum, exteriorize the fully mobilized stomach through the small incision, transect the stomach tissue, leaving a residual stomach opening of approximately 3–4 cm on the lesser curvature side. Perform full-thickness sutures at both ends of the residual stomach opening for traction.

After reconfirming the resection margin, insert the 25 mm circular-stapler through the central hole of the MSC and into the residual stomach from the opening on the lesser curvature side. Rotate the central shaft of the stapler to protrude its tip from the greater curvature side of the closed residual stomach. Insert an appropriately sized and trimmed T- tube (coated with paraffin oil) into the head of the circular stapler (Figures 3F,f). Rotate the central shaft back, place the circular stapler into the abdominal cavity via MSC, and re-establish pneumoperitoneum. Under laparoscopic visualization, rotate the central shaft of the stapler to remove the T- tube (Figures 3G,g). Connect the central shaft to the anvil on the duodenal stump, adjust the angle of the residual stomach-duodenum anastomosis using traction sutures on the residual stomach, and secure the anastomosis by tightening and firing the stapler (Figures 3H,h). This completes the laparoscopic anastomosis of the residual stomach and duodenum, which can be reinforced with 3-0 absorbable sutures under laparoscopic guidance. Remove the circular-stapler and T- tube through the central hole of the sealing cap.

Finally, close the opening on the lesser curvature side of the residual stomach using a linear cutting stapler, and reinforce the closure with 3-0 barbed sutures (Figures 3I,i).

### Postoperative management

On the third postoperative day, 100 ml of warm water was administered via mouth. If no significant abnormalities were observed, the amount of drinking water gradually increased. All patients were allowed to consume soft foods after their first bowel movement. Before discharge, all patients underwent a water-soluble contrast study to evaluate the gastroduodenostomy.

### Follow-up protocol

Patients were asked to undergo follow-ups every three months during the first year post-surgery, which included physical examinations, tumor marker tests, and imaging examinations. If no discomfort was reported, an endoscopic examination was conducted at the end of the first year.

## Results

The clinicopathological characteristics and surgical outcomes of the patients are shown in Table 1. The average surgical time was 143.8 ± 41.4 min. All patients had negative distal resection margins, and none of the patients underwent additional laparoscopic anastomoses or open surgeries. The clinical stages (cTNM) of the patients are IA (10)/IB (29)/IIA (6)/IIB (2), and the pathological stages (pTNM) are IA (8)/IB (22)/IIA (14)/IIB (3). All anastomoses were well-approximated and intact without evidence of leakage or structural compromise, and no anastomosis stenosis was found during long-term follow-up. The surgery demonstrated satisfactory early postoperative outcomes, with only two patient experienced postoperative complications, gastroparesis and pneumonia, and no other complications such as bleeding, pancreatitis, or pancreatic fistula. The patient recovered smoothly after conservative treatment. During the median follow-up period of 21.4 months (range: 13–34 months), no other gastroduodenostomy-related complications. No deaths occurred during the perioperative period or within the follow-up period.

**Table 1:** Clinicopathologic characteristics and operative results of the patients.

Variables	TLDG(*N* = 47)
Demographic data	
Male, female, *n* (%)	32 (68.1), 15 (31.9)
Age (years), mean ± SD	63.2 ± 7.3
Body mass index (kg/m^2^), mean ± SD	23.8 ± 3.5
Operative results	
Total operation time (min), mean ± SD	148.3 ± 41.4
Blood loss (ml), mean ± SD	102.5 ± 81.3
Postoperative recovery	
Mortality, n	0
Complications, *n* (%)	2 (4.3)
Gastroparesis	1 (2.1)
Pneumonia	1 (2.1)
Postoperative hospital stay, d, median (range)	9.6 (7–21)
First fatus, d, mean ± SD	3.6 ± 1.1
Pathologic results	
Tumor diameter(cm), median (range)	2.2 (1–3)
No. of retrieved lymph nodes, median (range)	20 (16–36)
Distal margin (cm), median (range)	2.6 (2.1–5)
Depth of invasiona T1/T2, *n* (%)	24 (51.1)/23 (48.9)
Clinical stages (cTNM)	IA(10)/IB(29)/IIA(6)/IIB(2)
Pathological stages (pTNM)	IA(8)/IB(22)/IIA(14)/IIB(3)
Follow-up, month, median (range)	21.4(13–34)

## Discussion

With the advancement of surgical instruments and techniques, numerous innovative approaches have been devised by international gastric surgery experts. Comparatively, the circular anastomosis reconstruction using a gastroduodenal tubular anastomotic device remains the simplest and most standard method in traditional open surgery. However, the difficulties encountered in laparoscopic duodenal purse-string suturing or creation, coupled with the lack of suitable surgical instrument channels, have led some surgeons to opt for the technical approach of gastric-duodenal anastomosis using a linear cutting stapler.

In 2002, Kanaya et al. first reported the Delta anastomosis, which involved the use of a linear stapler to perform a functional end-to-end Billroth I anastomosis between the remnant stomach and the posterior wall of the duodenum entirely under laparoscopy (10). This method was safe and feasible, with no significant postoperative dumping syndrome or residual gastritis reported. Patients had good eating habits and achieved a better quality of life (11). Tanimura reported in 2008 on the triangulating stapling technique (TST), which was an improvement upon the Delta anastomosis (12). This method was suitable for patients with smaller residual stomach volumes due to its reduced anastomotic tension. However, it carried an increased risk of abdominal cavity infection due to the potential spillage of gastrointestinal contents during the procedure. To address this, Omori et al. reported in 2014 on the modified intracorporeal triangulating anastomotic technique (INTACT) and its application in single-incision laparoscopic distal gastrectomy. The advantage of this technique lies in its ability to remove the stomach, duodenal stump, and staple line of the common opening using a linear stapler after closure, thereby enhancing safety by eliminating ischemic tissue (13).

In 2017, the Chinese team led by Hankun Hao reported the application of self-traction and post-disconnection Delta anastomosis (Delta SPLT) in totally laparoscopic distal gastrectomy (14). By tying a rope at the pylorus, the stomach was disconnected and the distal stomach was tractioned towards the left lower abdomen. A linear stapler was placed between the greater curvature of the residual stomach and the duodenal bulb to perform a side-to-side anastomosis between the posterior wall of the residual stomach and the long axis of the duodenum. The common opening was then closed, and the distal stomach was resected to form a triangular anastomosis. The number of stapler lines at the anastomotic site was reduced compared to the Delta anastomosis. Through a small sample study, it was found that this surgical technique can save on the use of linear staplers, simplify the operation process, and has a similar incidence of related complications to the Delta anastomosis (15).

In 2017, Japanese scholar Fukunaga invented the Billroth I-ART (augmented rectangle technique) anastomosis, which utilizes three linear staplers to perform a linear anastomosis entirely through laparoscopy, resulting in a rectangular anastomotic site. This ensures a wide anastomotic opening that is resistant to distortion and stenosis, facilitating the easy passage of food. Additionally, the technique eliminates the need for manual suturing, as the reconstruction of the digestive tract can be accomplished solely through the use of linear staplers, simplifying the procedure under total laparoscopy (16).

These surgical procedures also have corresponding disadvantages and limitations. All of them involve the use of a linear stapler, which requires mobilizing a sufficient length of the duodenal stump. This may result in excessive tension at the anastomotic site, poor blood supply, or anastomotic distortion, thereby increasing the risk of complications such as anastomotic leakage after surgery. These methods are not suitable for larger tumors or tumors close to the pylorus.

In this study, we utilized Lap-PSC in conjunction with MSC to perform a simple circular-stapled gastroduodenostomy, employing the same technique as in open surgery. This technique was first used in China and offers multiple advantages. Firstly, the excellent coordination between Lap-PSC and MSC has been successfully implemented by our team for several years in totally laparoscopic total gastrectomy, with over 100 surgical cases and a rich foundation in fully laparoscopic circular anastomosis. The MSC effectively provides a direct access channel to the abdominal cavity for the Lap-PSC through its central opening, preventing gas leakage. After completing lymph node dissection, a 5 cm small incision is made in the left upper abdomen to allow direct observation and palpation of the gastric tumor through the small incision before stomach resection, thereby directly determining the tumor location and resection margin. This combines the advantages of both open surgery and laparoscopically assisted surgery in terms of better judging the proximal resection margin. The incision was designed in the left upper abdomen, allowing for specimen extraction through the surgical incision while utilizing the MSC technique to introduce a circular stapler for gastro-duodenal anastomosis via the left upper abdominal approach, which better aligns with ergonomic principles and anatomical considerations. The Lap-PSC through the MSC allows for easy and simple operation to create a purse-string suture, similar to the operation of purse-string suturing instruments used in open surgery. The purse-string suture formed using Lap-PSC is uniform, symmetrical, regular, and highly reproducible. Based on our team's experience, laparoscopic surgeons with basic laparoscopic skills can easily and safely perform surgeries using Lap-PSC combined with MSC. The circular-stapler can easily and smoothly pass through the MSC without gas leakage. The anastomosis process is completed laparoscopically, offering a visual advantage over open surgery and laparoscopically assisted procedures. Additionally, since the anastomotic site of the residual stomach is located at the distal end of the greater curvature of the residual stomach, it can minimize the impact of anastomotic tension. The enrolled surgical cases primarily comprised patients with relatively early-stage disease. For cases involving larger tumors or more extensive gastric resection, Billroth II anastomosis was preferentially performed. Moreover, there is no need to consider the issue of ischemic lines on the posterior wall of the residual stomach during circular-stapled anastomosis, reducing anastomotic-related complications. This overcomes the difficulties and limitations caused by the small auxiliary incision during laparoscopically assisted Billroth I anastomosis. However, this study has several limitations. As a single-center retrospective study, the case accumulation remains in its early stages. No control group was established during the initial study design. In the absence of a control group, data analysis primarily focused on describing perioperative outcomes and postoperative complications in patients subjected to this novel technique, followed by descriptive statistical analysis. Our team plans to conduct prospective cohort studies with appropriately matched control groups in subsequent research. We believe these prospective controlled studies will provide higher-level evidence-based medical support for this surgical approach. Although our experience is limited, this study has achieved satisfactory early results in terms of simplicity, feasibility, and safety.

In summary, the TLDG with circular-stapler for Billroth I anastomosis combines the advantages of tactile feedback from open surgery and the minimally invasive benefits of laparoscopic surgery. This method may better align with the traditional concepts and habits of gastric and duodenal anastomosis among clinicians in China. Utilizing Lap-PSC and MSC in appropriately selected patients, especially in early-stage patients, TLDG with a circular-stapler for Billroth I anastomosis has been proven to be a safe and feasible surgical anastomosis approach.

## Data Availability

The original contributions presented in the study are included in the article/Supplementary Material, further inquiries can be directed to the corresponding author.
